# The how and why for multiple forms of hippocampal LTP

**DOI:** 10.3389/fnsyn.2026.1855059

**Published:** 2026-07-10

**Authors:** Christine M. Gall, Aliza A. Le, Gary Lynch

**Affiliations:** 1 Department of Anatomy and Neurobiology, University of California, Irvine, Irvine, CA, United States; 2 Department of Neurobiology and Behavior, University of California, Irvine, Irvine, CA, United States; 3 Department of Psychiatry and Human Behavior, University of California, Irvine, Irvine, CA, United States

**Keywords:** CA1, dentate gyrus, endocannabinoid, episodic memory, lateral perforant path, synaptic plasticity

## Abstract

Activity-induced Long-Term Potentiation (LTP)—characterized as it is by rapid induction, synapse specificity, and remarkable persistence—has long been considered a primary substrate for memory encoding. There has however been considerable debate about the cellular mechanisms responsible for producing the potentiated state. It seems that much of the confusion can be traced to an implicit assumption that there is a single form of stable potentiation. However, features of LTP in Schaffer-commissural (SC) input to CA1 and perforant path projections from entorhinal cortex to dentate gyrus (DG), show that different nodes of the hippocampal circuit express different types of LTP and that distinctions can be found even within the same population of synapses. For the SC system, brief theta burst stimulation (TBS) elicits LTP that is expressed by an expansion of the spine, postsynaptic density and associated AMPA receptor pool, and stabilized by reorganization of the actin cytoskeleton. Both sexes employ these mechanisms but females rely on locally synthesized estrogen and synaptic estrogen receptors to set processes in motion whereas males more heavily rely on metabotropic NMDA receptor signaling. Extended theta burst trains, high frequency stimulation (HFS), and spike timing paradigms engage mechanisms of LTP induction that differ from those produced by the minimal TBS. And an even more radical form of potentiation is expressed at lateral perforant path (LPP) connections with the DG. In this case, LTP is triggered postsynaptically but expressed presynaptically by increased transmitter release with an endocannabinoid providing the requisite retrograde messenger. These sex- and region-specific differences in plasticity have meaningful consequences for episodic memory encoding and vulnerability to neurological insults.

## 1 Introduction

The discovery of Long-Term Potentiation (LTP) (Bliss and Lomo, 1973; Bliss and Collingridge, 1993) suggested an answer to the question of how memories are encoded, an issue that had long been recognized as one of the great unresolved problems in the life sciences. LTP was quickly shown to possess two demanding features of a memory substrate: rapid induction and extreme persistence. Early work also showed that LTP is synapse-specific (Lynch et al., 1990) and thus consistent with the enormous capacity required for everyday, incidental forms of memory. A steadily accumulating body of evidence then established that LTP has a memory-like consolidation period (Lynch et al., 2007) and occurs during learning (Roman et al., 1987; Doyere and Laroche, 1992; Whitlock et al., 2006; Fedulov et al., 2007; Cox et al., 2014), and that blocking the effect disrupts encoding (Morris et al., 1986; Morris, 2003; Abraham et al., 2019). These observations resulted in a near consensus that LTP is indeed essential for storage of many types of information. But controversies soon emerged about the cell biological mechanisms responsible for the activity-driven increases in synaptic strength—certainly the most intense of these arguments involved the locus of the pertinent changes. The discovery papers (Bliss and Lomo, 1973; Bliss and Collingridge, 1993), which studied the perforant path projection to the dentate gyrus (DG), suggested that potentiation is expressed by an increase in transmitter release (Bliss et al., 1987) but later work on the Schaffer-commissural (SC) connection from field CA3 to CA1 obtained evidence for adjustments to the postsynaptic compartment without indications of changes in release (Muller and Lynch, 1988, 1989; Bliss and Collingridge, 1993; Manabe and Nicoll, 1994; Granger and Nicoll, 2014). The arguments on pre- vs. post-synaptic modes of action were soon joined by disputes over which of a bewildering array of cell signaling events are essential for shifting connections into the potentiated state (Kauer et al., 1988; Malenka et al., 1989; Lynch, 2003; Nicoll, 2003). This state of affairs has continued into the present.

Much of this confusion can, with the benefit of hindsight, be attributed to a failure to recognize that there might in fact be more than one type of LTP (Gall et al., 2024). That this idea was not widely embraced at some point during the early evolution of the field may have been due to the singular and rather unexpected nature of the studied phenomenon. It seemed unlikely that dramatic and months-long changes to synapses elicited by naturalistic patterns of synaptic activity would have evolved multiple times. Yet this does appear to be the case. As reviewed here, recent studies point to the following conclusions:

- Both pre- and post-synaptic varieties of LTP are present in the hippocampus;
- there are pronounced regional differences in the types of stable potentiation;
- the same synapses can express mechanistically distinct forms of LTP;
- and, equally surprisingly, males and females differ with regard to LTP induction mechanisms.

In the end, then, much of the debate about the nature of LTP can be explained by the high probability that the various investigators were studying different phenomena. But this conclusion brings with it a new question: Why do connections in the hippocampus and presumably the rest of cortical telencephalon follow different routes to reach what appears to be a common endpoint? A reasonable explanation might be that variations in the induction and expression of synaptic potentiation are needed to accommodate different forms of memory. Evidence in favor of this possibility is found in recent reports showing that sex differences in field CA1 LTP are associated with male vs. female advantages in the acquisition of the three basic elements of episodic memory (Le et al., 2024a). This idea will be discussed along with another, quite different explanation for the multiple forms of LTP expressed within hippocampus.

The present report will consider the above issues as evident in the induction and early expression of potentiation in the SC innervation of CA1 apical dendrites and perforant path input to the DG, arguably the two most intensively studied connections in hippocampus and the targets of much of our own work. Consideration of potentiation expressed by these two systems, which represent the major output and input stations of the hippocampal circuit, illustrates each of the points of diversity noted above. Moreover, analyses of the SC projections have shown that even for one population of synapses there are variations in the neurobiological mechanisms that give rise to LTP, with differences largely reflecting sex and induction parameters. These materials and the present discussion show that the long and sometimes tortuous history of synaptic plasticity has led to intriguing and satisfying explanations for what had once been a deeply mysterious though vital brain operation.

Given the present focus, we refer the reader elsewhere for descriptions of distinct forms of synaptic plasticity expressed by other hippocampal systems including the mossy fiber innervation of CA3 (Mellor and Nicoll, 2001; Kwon and Castillo, 2008), mossy cell innervation of the DG inner molecular layer (Hashimotodani et al., 2017; Gall et al., 2024), and input to CA2 pyramidal cells (Zhao et al., 2007; Carstens and Dudek, 2019; Oliva et al., 2023) as three important examples [see Gall et al. (2024) for review]. Moreover, for very broad review of mechanisms of plasticity in hippocampus including protein synthesis-dependent mechanisms of consolidation, we refer the reader to the presentation by Bliss et al. in The Hippocampus Book (Bliss et al., 2025).

## 2 Theta burst stimulation and Schaffer-commissural LTP

During learning, neurons in the cortical telencephalon commonly fire in short, high-frequency bursts separated by the period of the theta rhythm (Otto et al., 1991). The discovery that stimulation that mimics this pattern—short bursts of 100 Hz stimulation with theta frequency spacing—is near optimal for potentiating the CA3 to apical CA1, SC projections (Larson et al., 1986), provided important support for the argument that LTP is a memory substrate. Variants of this TBS paradigm have since found broad application in experimental and clinical work including transcranial and deep brain stimulation in humans (Ackerley et al., 2010; Hsu et al., 2011; Huang et al., 2011; Kouvaros and Papatheodoropoulos, 2016; Lu et al., 2024; Tao et al., 2024; Terao and Kodama, 2025). Moreover, responses to TBS have proven to be exceptionally informative in efforts to identify the cell biological mechanisms that shift synapses into their potentiated state (Larson and Munkacsy, 2015; Kouvaros and Papatheodoropoulos, 2016). Such work eventually led to explicit models for LTP substrates in the SC system, arguably the most extensively studied projection system in forebrain. However, as discussed below, more recent results call for significant revisions to these arguments and, in particular, the appreciation that there are diverse forms of SC LTP including clear differences between males and females, across the septotemporal axis of hippocampus, and contingent upon the nature of the inducing stimulation. We will begin with consideration of mechanisms of SC LTP induction and early stabilization in mid-septotemporal hippocampus in adult male rats and mice.

### 2.1 Molecular substrates for postsynaptic induction and initial expression of SC LTP

**Figure 1:**
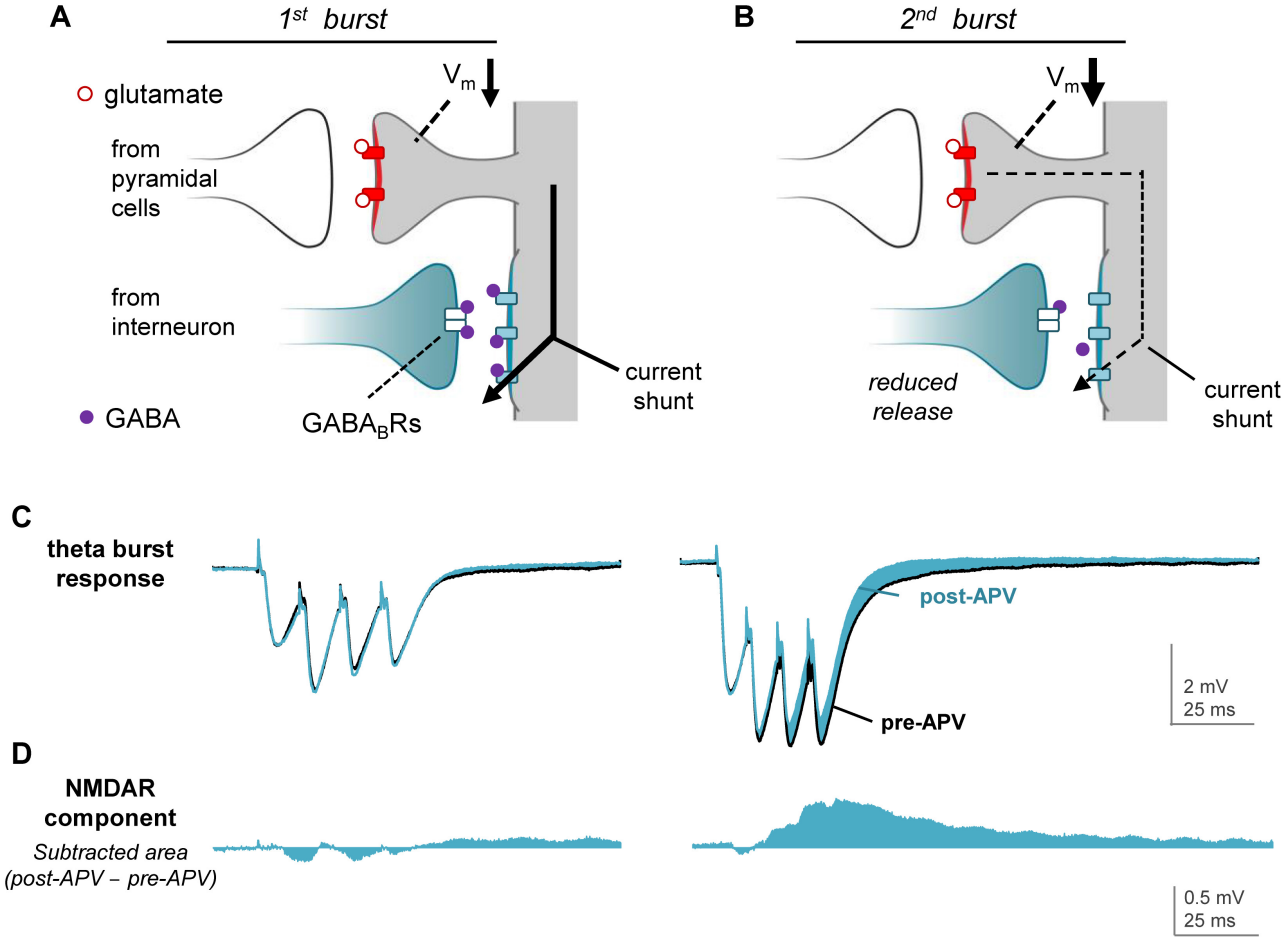
Theta burst stimulation (TBS) entails a transient shunting of inhibition and enhanced NMDAR activation. **(A)** Schematic showing the CA3 to CA1 pyramidal cell synapse (gray, postsynaptic) and input from a local interneuron (blue) activated by CA3 input. The inward current from the glutamatergic synapse during a first burst reduces the membrane voltage (*V*_m_) in the innervated spine but this effect is lessened by shunting of the current via the slightly delayed activation of neighboring GABAergic synapses. GABA released during the burst binds to presynaptic GABA_B_Rs on the interneuron terminal. **(B)** The GABA_B_Rs produce a gradually developing inhibition of release of GABA from the interneuron terminal. As a result, with a 2nd theta burst arriving 200 ms after the 1st, lower levels of GABA are released, thereby minimizing the shunt, and the excitatory current on the spine produces a larger depolarization. **(C)** Field EPSP recordings of responses elicited by the 1st and 2nd theta bursts: the amplitude and duration of the 2nd response are increased over the 1st response, as expected for relaxed feedforward inhibition. **(D)** Isolation of responses sensitive to infusion of an NMDAR antagonist (APV) shows that the NMDAR response is small during TBS #1 and substantial during TBS #2. APV has its largest effects during the 3rd and 4th fEPSP on the second burst response. C and D are adapted from Le et al. (2024a).

Studies from a number of laboratories have described LTP substrates for SC contacts in CA1 stratum (str.) radiatum. This SC potentiation, triggered by a conventional train of 10 theta bursts, develops quickly, is specific to the synapses directly activated by TBS and can persist for hours in hippocampal slice preparations and weeks *in vivo* (Staubli and Lynch, 1987; Abraham et al., 2002; Abraham, 2003; Lynch et al., 2013; Nicoll, 2017). It also has a memory-like consolidation period lasting from 10 to 30 min post-TBS during which it becomes progressively more resistant to disruption (Huang and Hsu, 2001; Abraham et al., 2002; Lynch et al., 2007; Bramham, 2008; Lynch et al., 2013). It is now well-established that, for this system, induction of the potentiated state occurs in the postsynaptic compartment with activation of the voltage-dependent NMDA receptors (NMDARs) and associated calcium influx being critical initiating steps (Coan et al., 1987; Larson and Lynch, 1988; Bliss and Collingridge, 2019). The theta burst interval (150–200 ms between bursts) allows interneuron terminals to engage GABA_B_ autoreceptors and thereby transiently reduce shunting inhibition—this causes a marked enhancement of the composite (depolarizing) response to a second theta burst and activation of voltage-sensitive NMDARs (Figure 1) (Larson and Lynch, 1988; Davies and Collingridge, 1996; Collingridge, 2026).

**Figure 2:**
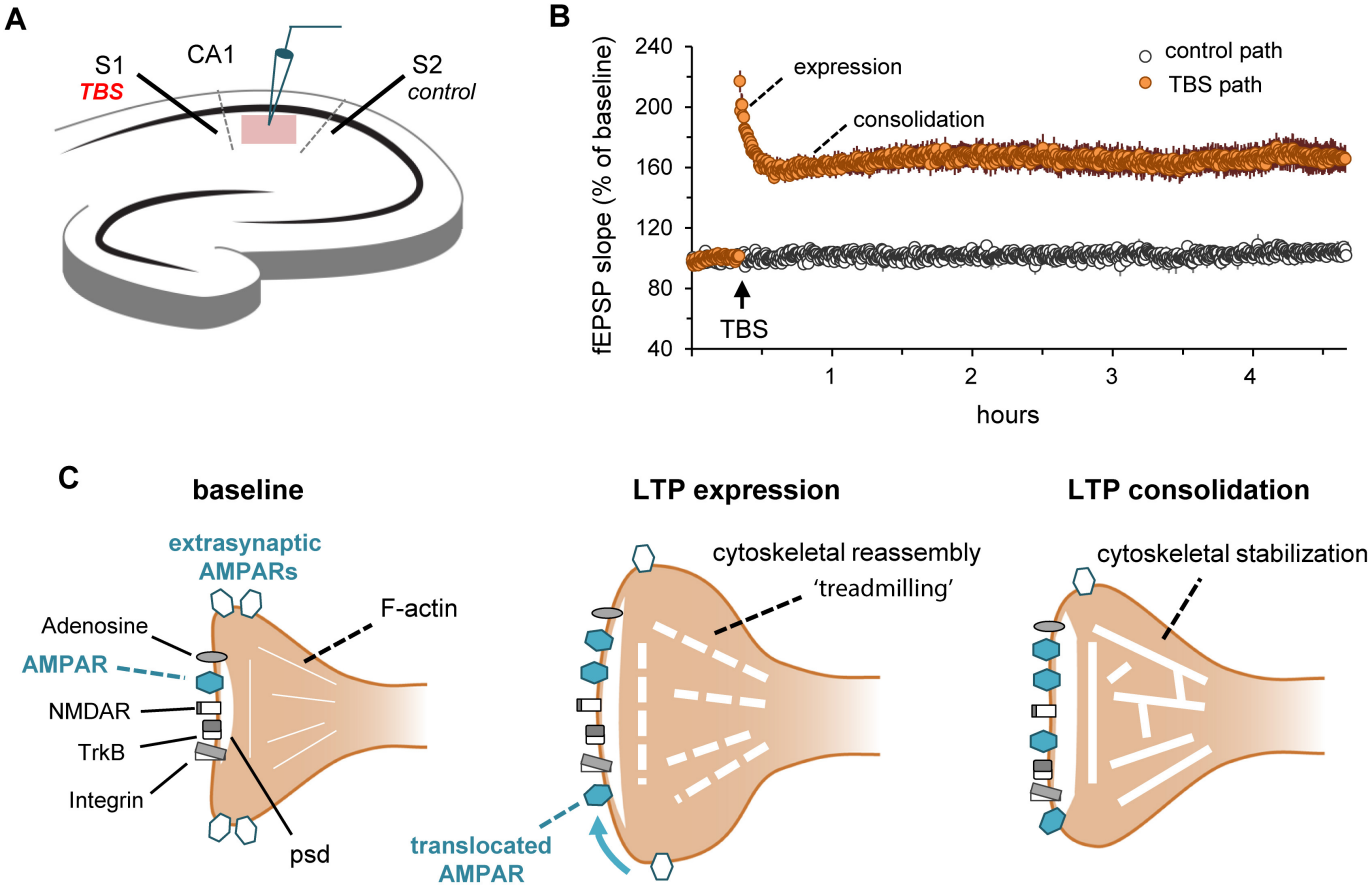
TBS-induced SC LTP entails spine and psd expansion, and cytoskeletal mechanisms for consolidation. **(A)** Conventional acute hippocampal slice arrangements with two stimulating electrodes (S1, S2) activating independent inputs to the same CA1 apical dendritic field (pink box). **(B)** Plot of fEPSP responses shows that a single 10 burst TBS train applied via S1 (orange) elicits pronounced facilitation within 5 s, that decays to a steady plateau over 10 min and is sustained without decrement for 4 h. Continued 0.05 Hz stimulation of the control axons (open symbols) shows no change over time. **(C)** Illustration of a CA1 dendritic spine showing (at left) elements known to be critical for producing LTP, including membrane receptors for glutamate (NMDAR, AMPAR), BDNF (TrkB), adenosine, and matrix ligands (integrins). **(Middle)** In the minutes following TBS initial LTP expression involves expansion of the spine and postsynaptic density (psd), an addition of AMPARs to the active zone via insertion and migration from extrasynaptic sites, and new actin polymerization leading to higher F-actin levels in the spine head. The new actin filaments are, for a time, dynamic with actin being added to and subtracted from newly formed filaments. The potentiated state is readily reversible during this period by low frequency stimulation or activation of adenosine A_1_ receptors as two examples. **(Right)** Initial LTP consolidation involves an additional set of signaling pathways that stabilize the reorganized actin cytoskeleton. Panel B is adapted from Lynch et al. (2013). Not shown: later phases of consolidation including protein synthesis dependent events (Abraham and Williams, 2008).

Diverse lines of evidence indicate that for the SC system, stable adjustments that increase the size of the synaptic response—LTP expression—are also postsynaptic. First, potentiation involves an increase of AMPA receptor (AMPAR)-gated currents without a change in responses mediated by colocalized NMDARs (Kauer et al., 1988; Muller et al., 1988), hence there is a marked change in the NMDAR/AMPAR ratio. This dissociation argues strongly against an increase in transmitter release. Second, SC LTP requires an increase in postsynaptic calcium (Lynch et al., 1983; Malenka et al., 1988, 1989) and associated activation of calcium/calmodulin-dependent kinase II (CaMKII) (Lisman et al., 2012). Third, as induced with a short, naturalistic TBS train, SC LTP does not entail an increase in release probability (Muller and Lynch, 1988; Manabe et al., 1993) but is associated with an enlargement of dendritic spines (Lee et al., 1979; Wang et al., 2008; Stein et al., 2021) and the postsynaptic specialization (Chen et al., 2007), thereby enabling an increase in the size of the AMPAR pool (Shi et al., 1999) (Figure 2).

Increases in AMPAR-mediated currents with SC LTP have been associated with movement of additional AMPARs into the synaptic membrane, either by membrane insertion or lateral diffusion from perisynaptic regions (Collingridge et al., 2004). Recent work suggests that the increases in evoked AMPAR currents with LTP may also reflect redistribution of the receptors within the active zone. Work using super resolution and expansion microscopy has identified protein complexes that define subsynaptic domains (SSDs) associated with presynaptic vesicle release and the postsynaptic response (Tang et al., 2016). These analyses indicate that LTP may increase the alignment of the SSDs to form nanocolumns that enhance the postsynaptic response by placing points of release immediately adjacent to concentrations of AMPARs (Hruska et al., 2018; Xu et al., 2025).

Overall, the above findings have given rise to the broadly accepted scheme whereby TBS-induced SC LTP involves time-locked activation of NMDARs followed quickly by structural changes to the target synapses leading to greater numbers of AMPARs being engaged by a given amount of released glutamate (Maren et al., 1993; Hayashi et al., 2000; Lynch et al., 2007; Granger and Nicoll, 2014; Diaz-Alonso and Nicoll, 2021).

The discovery that newly induced LTP can be erased by low frequency activation of the potentiated synapses (Barrionuevo et al., 1980; Huang et al., 2001; Kramar et al., 2002) showed that the above noted induction and expression events are followed by slower stabilization (consolidation) processes. The limited period during which recently induced LTP can be erased by this and other manipulations (adenosine infusion, hypoxia, cooling) indicates that 10–30 min are needed to lock synapses into their potentiated state (McGaugh, 1966; Arai et al., 1990a,b; Bittar and Muller, 1993; Staubli and Scafidi, 1999; Huang et al., 2001). The fact that only a small percentage of synapses are engaged by TBS in LTP experiments (Chen et al., 2007) greatly complicated efforts to uncover the cell biological events underlying this consolidation. However, with a rationale (Lynch and Baudry, 1984) and evidence (Krucker et al., 2000; Okamoto et al., 2004) that filamentous (F) actin was critical for LTP stabilization, we returned to this issue and found that TBS elicits marked increases in F-actin in a small subset of spines as identified with *in situ* phalloidin labeling (Lin et al., 2005; Kramar et al., 2006), a result that agrees with others using different approaches (Okamoto et al., 2007) and induction with high frequency stimulation (HFS) (Ramachandran and Frey, 2009).

**Figure 3:**
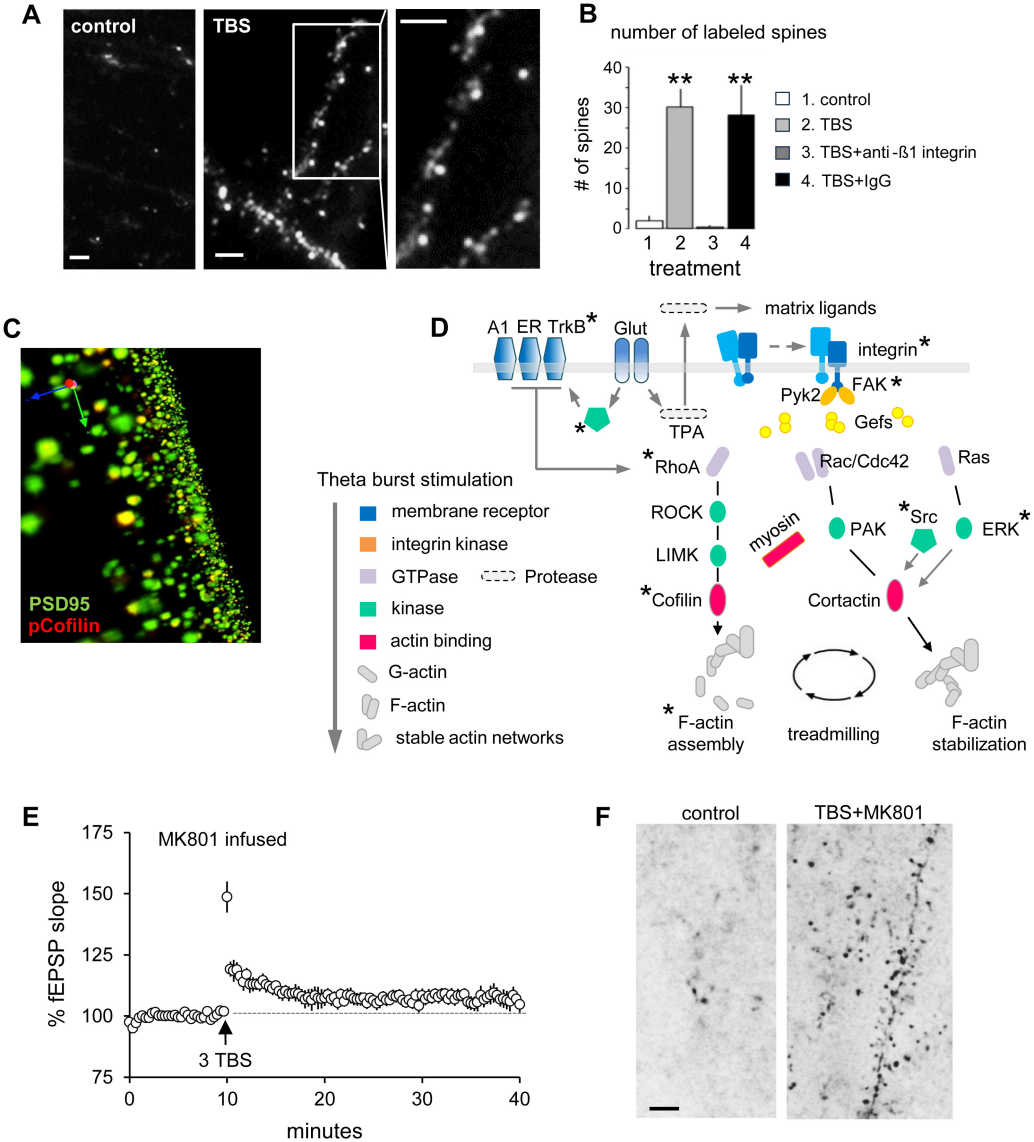
Molecular substrates of initial TBS-induced SC LTP consolidation including metabotropic NMDAR function. **(A)** Images show phalloidin labeling in CA1 SR in slices receiving control 0.05 Hz stimulation **(left)** or 10 burst TBS applied to SC afferents: TBS increases phalloidin labeling of newly formed filamentous (F) actin (white puncta) localized to dendritic spines. The increase in punctate labeling is blocked by Latrunculin A which prevents actin polymerization (not shown). **(B)** Numbers of F-actin enriched (phalloidin-labeled) spines under the indicated conditions: control, receiving 0.05 Hz SC stimulation; 10 burst TBS; TBS in the presence of ß1 integrin neutralizing antisera (anti-ß1) or control immunoglobulin (IgG). **(C)** Image shows a 3-D reconstructed Z-stack showing immunolabeling for PSD95 (green) and phospho (p) cofilin (red; yellow denotes dual labeling) and processed for Fluorescent Deconvolution Tomography (FDT), an approach used to quantify receptor activation and signaling activities engaged with TBS-induced SC LTP. The 3-D image was rotated to show labeling from the surface to depth of the tissue section (right to left). **(D)** Schematic illustration of postsynaptic receptors and signaling cascades that are engaged by TBS and control TBS-induced actin remodeling and LTP: note, signaling downstream from RhoA and Rac/Cdc42 control new F-actin polymerization and F-actin stabilization, respectively. Using FDT, we confirmed the critical involvement of each receptor type and signaling intermediary shown, in studies of TBS-induced SC LTP in slices from adult rats and mice. Identification of key signaling elements in females verified that each cascade was similarly critical for stable LTP but further showed that, in females only, TBS-activation of key elements (marked with asterisks) required intact estrogen/estrogen receptor signaling. (**E, F**) The NMDAR channel blocker MK801 eliminates LTP induced by 3 burst TBS **(E)** but not the TBS-induced increase in phalloidin-labeled (F-actin enriched) spines. Panels A and B adapted from Kramar et al. (2006). Scale bars = 5 μm.

Evidence for F-actin involvement led to work, using reconstruction of spines immunolabeled with state-specific antisera to receptors and signaling proteins, that quantified TBS-induced increases in signaling activities within specific neuronal compartments (Rex et al., 2009; Chen et al., 2010b). Combined with a variety of experimental manipulations, this approach revealed that in rats and mice of both sexes, SC LTP consolidation relies upon signaling to and reorganization of the subsynaptic actin cytoskeleton (Kramar et al., 2006; Chen et al., 2007; Rex et al., 2009; Le et al., 2024a) (Figures 2C, 3A–C) and, further, that activation of signaling proteins can be used to identify recently potentiated synapses (Chen et al., 2007). The pertinent signaling cascades have a great deal in common with those found at adhesion junctions throughout the body (Giancotti and Ruoslahti, 1999; Brakebusch and Fassler, 2003; DeMali et al., 2003; Danen et al., 2005). In particular, as illustrated on Figure 3D, these studies showed that in the SC system a single 10 burst train of TBS activates small GTPases (RhoA, Rac, Cdc42, Ras) within the postsynaptic compartment. Signaling downstream from RhoA (i.e., RhoA > RhoA kinase (ROCK) > LIMK > Cofilin) triggers actin polymerization followed by the expected “treadmilling” of newly formed actin filaments (Carlier, 1998; Kramar et al., 2006; Rex et al., 2010). Signaling from Rac/Cdc42 and Ras to Arp2/3 (Liu et al., 2024) contributes to the branching and stabilization of the modified actin network (Krucker et al., 2000; Rex et al., 2009, 2010; Borovac et al., 2018) (Figures 3C, D). These activities are largely driven by glutamate receptor activation but are also influenced by modulatory systems including the adenosine A1 receptor, which inhibits RhoA signaling but leaves that downstream from Rac intact (Rex et al., 2009), and estrogen acting through membrane variants of the canonical estrogen receptors to facilitate RhoA activation both alone and synergistically with TBS (Kramar et al., 2009).

Integrin adhesion receptors play a critical role in TBS-driven actin management and SC LTP consolidation (Chang et al., 1999; Hernandez et al., 2001; Kramar et al., 2006; Chan et al., 2007; Wang et al., 2016a) (Figures 3B, D). TBS activates postsynaptic, ß1-subunit containing integrins at SC synapses and, as shown in hippocampal slices, inhibition of ß1 integrin activation prevents SC LTP induced by TBS or HFS (typically, 1 or more trains of 100 Hz stimulation) in both sexes (Staubli et al., 1990; Huang et al., 2006; Kramar et al., 2006; McGeachie et al., 2011; Wang et al., 2016a). Curiously, synaptic ß1 integrins exhibit an activation-deactivation cycle: Following activation by one TBS train, there is a 1 h period during which integrin activation and LTP are refractory to a second TBS train (Kramar et al., 2006; Babayan et al., 2012), a phenomenon that may underlie effects of spaced stimulation on LTP magnitude discussed below.

Beyond reliance on integrin activation, TBS-induced actin remodeling and SC LTP depend on the neurotrophin BDNF and its TrkB receptor (Korte et al., 1998; Chen et al., 1999; Bramham and Messaoudi, 2005; Rex et al., 2007; Lu et al., 2008; Chen et al., 2010b). Among other activities (Minichiello, 2009), TrkB activates RhoA and Rac signaling to regulate the actin cytoskeleton (Rex et al., 2007; Hedrick et al., 2016) (Figure 3D). BDNF also stabilizes the WAVE regulatory complex at the synapse thereby increasing signaling to Arp2/3 which facilitates growth of the branched F-actin network and spine enlargement (Shohayeb et al., 2024). Although TrkB is present in both pre- and post-synaptic elements, TBS-induced TrkB activation was detected in the postsynaptic compartment only (Chen et al., 2010b). Interestingly, evidence suggests that TrkB interacts with synaptic integrins in support of LTP. Downregulation or antagonism of ß1 integrins blocks TBS-driven TrkB activation in adult rat and mouse hippocampus (Wang et al., 2016a, 2018b).

Together these findings describe a synaptic activity regulated system that uses multiple synaptic receptors and signaling through parallel GTPase cascades to remodel the actin cytoskeleton and junctional structure, and to thereby contribute to the consolidation of SC LTP (Figures 2, 3C, D).

It is worth noting that there are other modulatory substances that are at least in part conveyed to hippocampus via the circulation and influence synaptic plasticity. These include, but are not limited to, sex steroid and other hypothalamic hormones. The best documented example among this class is estrogen (which is both circulating and produced by neurons within hippocampus, hence termed a “neurosteroid”). As shown in Figure 3D, and discussed more fully below, infused estrogen activates RhoA. This facilitates actin polymerization and TBS-induced SC LTP (Kramar et al., 2009; Wang et al., 2016a) and, seemingly through the same mechanisms, potentiates baseline responses in the absence of LTP-inducing stimulation (Wang et al., 2018b; Jain et al., 2019). Other hormones (e.g., oxytocin) have been shown to influence SC LTP in different contexts (Chavez et al., 2026) but the mechanisms of action have not been resolved.

Finally, monoaminergic transmitters also influence, and largely facilitate SC LTP. Hippocampal slice studies have shown that infusion of monoamine receptor agonists themselves gives rise to a slow potentiation of SC responses, whereas activation of dopaminergic (Hansen and Manahan-Vaughan, 2014) and noradrenergic (Nguyen and Gelinas, 2018) receptors can enhance SC LTP most particularly as induced by HFS. Similar enhancement of LTP is observed in the DG (Harley et al., 2005; Hansen and Manahan-Vaughan, 2014, 2015), thereby suggesting that these influences are unlikely to account for mechanistic differences between the perforant path and SC forms of LTP.

Whether endogenous monoaminergic signaling is required for LTP induction is less clear, as antagonist studies have yielded mixed results *in vitro* and *in vivo*. Dopamine receptor antagonists disrupt synthesis-dependent stages of LTP in particular, as opposed to early protein synthesis independent events (Stramiello and Wagner, 2008; Lisman et al., 2011; Hansen and Manahan-Vaughan, 2014; Papaleonidopoulos et al., 2018). The effect of blocking β-adrenergic receptors on SC-LTP varies along the septotemporal axis of the hippocampus (see Section 2.2). Results for the DG are mixed: some support the view that noradrenaline receptors are required for LTP and others do not (Bramham et al., 1997; Swanson-Park et al., 1999). In both regions, observed modulatory actions are ascribed, like those of dopamine, to effects on protein synthesis and, thus, to later stages of LTP consolidation (Nguyen and Gelinas, 2018; Maity et al., 2020; Fuchsberger et al., 2025) and not to processes illustrated in Figure 3D. Regardless, given the strong influences of arousal and response to novelty on memory retention (Sara, 2009; Tse et al., 2023), analysis of these transmitter systems on the initial LTP consolidation machinery warrants further consideration.

### 2.2 Differences in SC LTP across the hippocampal septotemporal axis

Although signaling activities described above are associated with SC LTP across the septotemporal axis of hippocampus, there are regional differences in the stimulation threshold and magnitude of LTP in both CA1 and the DG with potentiation being less robust in temporal (ventral) planes. Thus, in adult rat hippocampal slices, stimulation with just 2 or 5 theta bursts was sufficient to elicit stable SC LTP (lasting over 2 h) in dorsal (septal) hippocampus whereas similar potentiation in ventral (temporal) hippocampus required 20 theta bursts (Kouvaros and Papatheodoropoulos, 2016). Moreover, the magnitude of SC LTP induced with a given level of stimulation (HFS or short theta trains) is reportedly greater in dorsal than in ventral hippocampus (Colgin et al., 2004; Maggio and Segal, 2007a,b; Kouvaros and Papatheodoropoulos, 2016).

These differences in the facility for SC LTP likely reflect differences in both afferents and neurochemical systems across the septotemporal axis of hippocampus [see (Strange et al., 2014) for review]. There are greater numbers of interneurons in more temporal fields suggesting greater local inhibition. Noradrenergic and dopaminergic inputs more densely innervate ventral as compared to dorsal fields and, although different serotonergic afferents target dorsal and ventral hippocampus, the overall density of 5-HT terminals is greater ventrally. Functional studies suggest that physiological levels of dopamine, acting through the D1/5 receptors increase LTP magnitude in both dorsal and ventral hippocampus, whereas agonists of the D1 receptor enhance LTP in ventral fields only (Papaleonidopoulos et al., 2018). Noradrenaline, serotonin (5-HT) and glucocorticoid receptors are greater in ventral vs. dorsal hippocampus as are levels of acetylcholine and choline acetyltransferase (McEwen, 2002; Strange et al., 2014). An exception to this trend is the greater concentration of adenosine A1 receptors in dorsal vs. ventral hippocampus. In contrast to these gradients in monoaminergic and, potentially, purinergic systems, levels of BDNF and its TrkB receptor reportedly do not differ across septotemporal planes (Toyoda et al., 2014).

### 2.3 Ion flux independent NMDAR functions support stabilization of male SC LTP

Recent studies suggest the need for some revisions to the above description of how TBS elicits stable SC LTP. The prevailing model posits that NMDARs activate calcium-dependent proteases (calpains) which disrupt the extant actin cytoskeleton and then enzymes and binding proteins promote entry of AMPARs into, and potentially shifting of AMPARs within, an expanded postsynaptic zone (Collingridge et al., 2004; Lynch et al., 2007; Diaz-Alonso and Nicoll, 2021; Collingridge, 2026). Subsequent stabilization of the new arrangements is provided by somewhat slower cytoskeletal modifications. Because competitive antagonists of NMDARs (e.g., APV) completely block actin signaling induced with short TBS (Lin et al., 2005; Rex et al., 2007; Le et al., 2024a), it was further assumed that calcium influx through the NMDARs is needed to set integrin- and TrkB-driven events, and thus actin remodeling, in motion. However, newer studies show that although blocking NMDAR-mediated currents with the channel blocker MK801 blocks SC LTP in adult hippocampal slices from both sexes, it has little if any effect on TBS-induced increases in spine F-actin (Le et al., 2024a) (Figures 3E, F). In accord with this, glutamate uncaging induced spine enlargement, considered a structural signature of LTP, is similarly reliant on NMDARs but not upon their ion-flux activity (Stein et al., 2021; Park et al., 2022). These results denote a remarkable state of affairs in which fully blocking NMDAR activation (by blocking glutamate binding) eliminates all evidence of synaptic modifications, whereas blocking NMDAR-mediated ion flux does not interfere with TBS-induced actin remodeling or spine enlargement: Thus, structural events that stabilize potentiation can be elicited in the absence of SC LTP expression.

A growing body of evidence suggests that ion flux independent (metabotropic) NMDAR signaling is largely mediated by the GluN2B subunit of the tetrameric receptor (Kessels et al., 2013; Li et al., 2022; Barnes et al., 2025). In line with this, we found that the GluN2B antagonist Ro25-6981 disrupts TBS-induced actin regulatory signaling, increases in spine filamentous (F) actin and LTP consolidation in hippocampal slices from adult male rats without interfering with the initial induction and expression of potentiation (Le et al., 2024a). Evidence that the same GluN2B antagonist blocks field CA1-dependent episodic, cue location memory in adult males indicates that the metabotropic NMDAR functions are critical for synaptic plasticity *in vivo* as well.

### 2.4 Evidence for metaplastic modulation of SC LTP: spaced trials effects and synaptic tagging

Additional modifications to the basic SC-LTP model were necessitated by the discovery that recent activity of the SC system can influence the likelihood that a given amount of stimulation will induce LTP. Such “metaplasticity” [i.e., activity-related changes in neuronal state that influence the facility for later LTP or long-term depression (LTD)] (Abraham and Bear, 1996; Hegemann and Abraham, 2021) is apparent in studies demonstrating that there are stimulation-spacing effects for SC potentiation that are akin to the spaced trials effect in learning. It has been known since the 19th century that many types of memory are more efficiently encoded when a given amount of training is distributed across multiple sessions rather than being administered in one massed trial in humans (Ebbinghaus, 1885; Cepeda et al., 2006; Kornmeier and Sosic-Vasic, 2012; Smolen et al., 2016) and for hippocampus-dependent learning in rodents (Seese et al., 2014; Nonaka et al., 2017; Tintorelli et al., 2020). Although early studies had shown that 20 theta bursts applied to the mid septotemporal SC system do not elicit more potentiation than 10 bursts (Larson et al., 1986; Kouvaros and Papatheodoropoulos, 2016), later work, using slices from adult Sprague Dawley rats, showed that after SC stimulation with an initial 10 burst TBS train (TBS#1), a second train (TBS#2) applied 1 h after the first doubles the magnitude of SC LTP (Kramar et al., 2012). In contrast, TBS#2 applied 10, 20, or 40 min after TBS#1 elicited no further potentiation (Kramar et al., 2012; Lynch et al., 2013). These results indicate that there is a refractory period after TBS#1 that is released 60 min later at which point additional stimulation will enhance LTP. Evidence for response enhancement with TBS trains spaced by 1 h was replicated by Harris and colleagues who further reported that this spaced stimulation effect was absent in prepubescent rats and that the refractory period following TBS#1 was longer after very strong initial stimulation (i.e., after 8 TBS trains as compared to a single TBS train) (Cao and Harris, 2014).

The above studies provide circuit level evidence for the efficacy of spaced stimulation but did not determine if a post-potentiation refractory period, and further response enhancement by delayed stimulation, are exhibited by individual synapses. This issue was addressed by Flores et al. (2025) using local high frequency glutamate uncaging (HFU) to evaluate effects on spine size, a structural correlate of LTP. They found that after an initial HFU-induced potentiation of individual spines, those same spines were refractory to further potentiation with HFU#2 applied 30 and 45 min later. However, this refractory state was released after 60 min, at which time a second round of HFU elicited further spine enlargement (Flores and Zito, 2024). The refractory period was spine-specific (i.e., not evident for adjacent spines on the same dendrite) and was associated with both reduced CaMKII activation and an absence of HFU-induced membrane GluA2 insertion relative to robust measures in control spines. Although doubling the intensity of HFU#2 at 30 min increased the level of CaMKII activation somewhat (relative to effects of the weaker initiating stimulation) the increase in spine size was still not statistically significant indicating that processes other than CaMKII activity define the refractory period wherein the capacity for potentiation is reduced.

In our studies, the efficacy of spaced SC stimulation seemed to be related to the unusual, activation-deactivation behavior of ß1-integrins (Babayan et al., 2012). As noted above, these adhesion proteins have potent effects on actin regulatory machinery and the signaling activities of other synaptic receptors (Wang et al., 2008, 2016a). At SC synapses, the ß1 integrins dropped out of their active state by 10–15 min after TBS#1 and neither ß1 integrins nor their signaling intermediary focal adhesion kinase, could be activated by TBS#2 until 1 h later (Babayan et al., 2012). Unexpectedly, blocking these integrins at the 1 h time point erased the initial potentiation, thereby revealing a second and considerably delayed LTP consolidation stage. Similarly, blocking ß1 integrins in field CA1 at 1 h after initial cue exploration in the object location memory test eliminated retention of cue location. Thus, it appears that recovery of integrin responsivity is associated with consolidation of initial LTP and memory encoding, as well as with the delayed emergence of a capability for further acquisition.

Regarding spaced stimulation, it is noteworthy that other investigators observed spaced stimulation effects with repeated trains of HFS spaced by 5–10 min, and showed this approach recruits signaling that is not evident with a single bout of HFS (Nguyen and Woo, 2003; Woo et al., 2003; Blitzer et al., 2005). Thus, the presence of a clear spacing effect, that entails a post-induction refractory period, and set period for the release of the refractory state, may be a feature of SC LTP induced with more naturalistic stimulation. Or, it may be the case that the timing by which response enhancement by spaced stimulation can be realized reflects the intensity of the initial inducing stimulation (Cao and Harris, 2014).

These spaced stimulation effects on SC LTP share some conceptual similarities with the synaptic tagging and capture (STC) hypothesis wherein the plasticity inducing stimulation is proposed to set a tag on a set of activated synapses (the first stage of plasticity, be it LTP or long-term depression, LTD) and then this tag “captures” plasticity-related proteins (PRPs) for the final stabilization of the change in synaptic strength (Redondo and Morris, 2011; Okuda et al., 2021; Bin Ibrahim et al., 2024). In this scheme the protein synthesis dependence of LTP consolidation reflects the need for production of PRPs, and that production could occur either before or after the stimulation that set the synaptic tag. Many candidate PRPs have been identified (Okuda et al., 2021), including kinases associated with actin reorganization and stabilization, but events following capture would be later stage, protein synthesis dependent processes relative to mechanisms considered here. For fuller consideration of these processes, and their consequences for learning we refer the reader to recent published works (Okuda et al., 2021; Bin Ibrahim et al., 2024; Koek et al., 2024b).

## 3 Distinct forms of SC plasticity

### 3.1 Initial stages of TBS-induced SC LTP include overlapping decremental and stable variants

**Figure 4:**
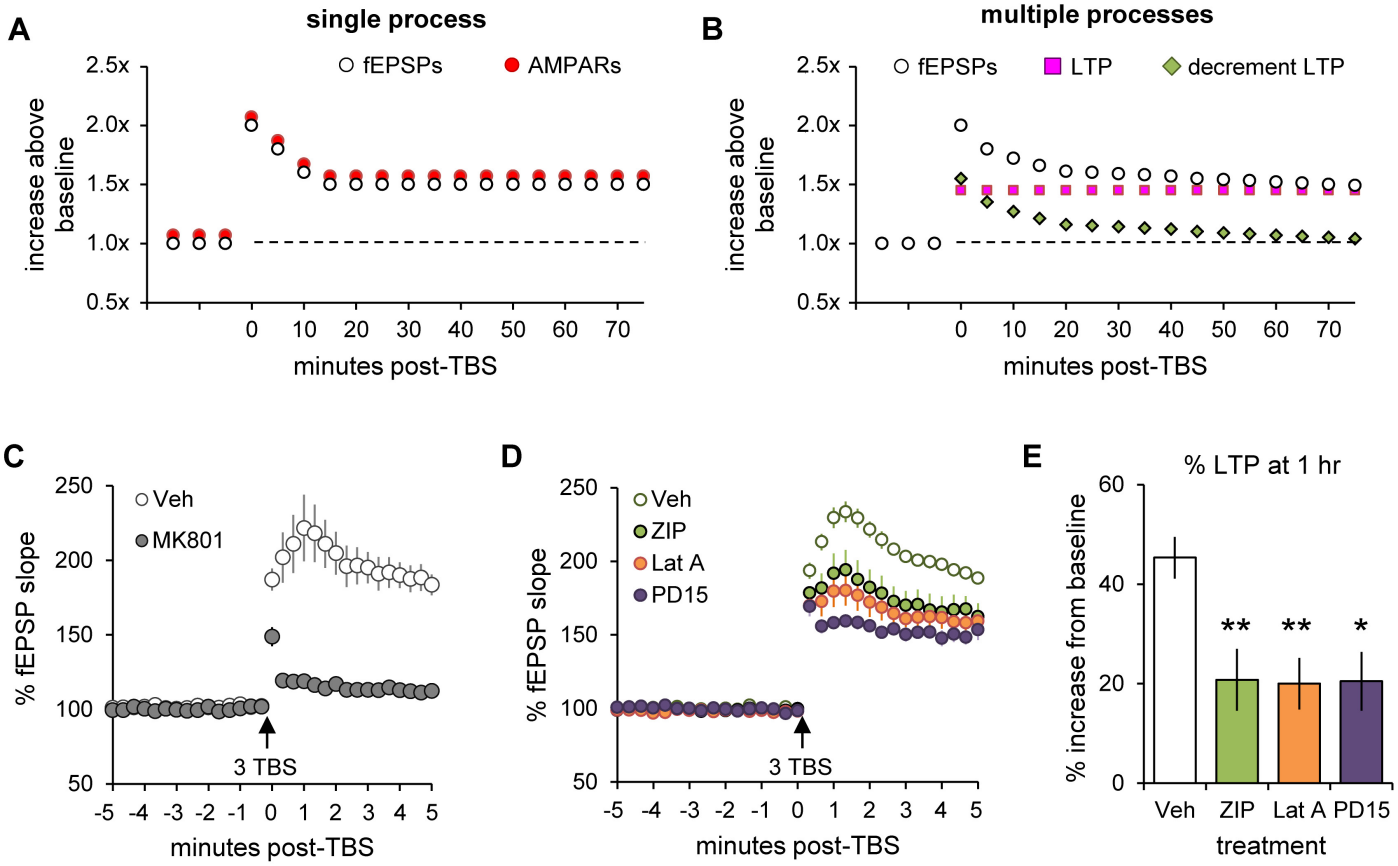
Evidence for multiple overlapping forms of potentiation in the initial stages of SC LTP. **(A)** A single process model wherein the expression of TBS-induced LTP is simply a function of changes in the number of postsynaptic AMPARs. **(B)** A multiple process model wherein one or more decremental form of potentiation (green) coexists with a stable LTP effect (pink): In such a case the observed post-TBS response (open symbols) would reflect a summation of the two processes and thus exhibit an initial decline in response to a stable plateau. (**C–E**) In adult mouse hippocampal slices, MK-801 (30 μM), Latrunculin-A (Lat A, 200 nM), ZIP (2 μM), PD150606 (PD15, 1 μM) or vehicle (Veh) were infused into the slice chamber for 30 min and then 3 burst TBS (3-TBS) was delivered and SC fEPSP recordings resumed for 1 h. **(C)** With Veh infusion synaptic responses grew during the first 2 min post-TBS; MK801 reduced but did not eliminate the increase in the first post-TBS response (TBS + 20 s). **(D)** The initial rising phase in the post-TBS response was markedly reduced by the PKMζ inhibitor ZIP, actin polymerization inhibitor Lat A and calpain I inhibitor PD15. Note that none of these drugs blocked facilitation of the first post-TBS response. **(E)** Each compound used in D caused a profound reduction but not elimination of LTP recorded at 60 min post-TBS (**p < 0.01, *p = 0.021).

The large potentiation elicited in CA1 by a single 10 burst TBS train (10-TBS) typically decays for about 10 to 15 min—a period sometimes referred to as Short-Term Potentiation (STP) (Bliss et al., 2025)—and then stabilizes at a plateau that is substantially higher than baseline (see Figure 2B; It is noteworthy that here we are referring to the transient decay in the amplitude of potentiation over the first 15 min and not a gradual loss of potentiation *per se*). Detailed pharmacological work has linked STP to NMDAR activation (France et al., 2022) but little is known about the substrates for STP and the extent to which they overlap those for LTP. Within the context of the model described in preceding sections, the simplest interpretation of the potentiation curve would involve a rapid expansion of the synaptic AMPAR population (initial LTP expression) that decays steadily over about 10 min, until being secured by the onset of events that arrest the decline and stabilize both the AMPAR pool and structural changes (i.e., LTP consolidation; Figure 4A). However, it is also possible that STP constitutes a form (or forms) of plasticity that are distinct from LTP with the STP curve reflecting one effect superimposed upon another (Figure 4B).

Recent studies using a minimal three theta burst (3-TBS) paradigm to induce SC LTP in mouse hippocampal slices showed that, following this short train, single pulse fEPSP responses in CA1 grow larger over the first 90 s (Chavez et al., 2026). The NMDAR channel blocker, MK801, prevents the response facilitation at 90 s but has a much smaller effect on the response recorded 20 s after 3-TBS (Figure 4C). This result indicates that even a very minimal amount of patterned afferent activity suffices to trigger a transient facilitation that is independent of NMDAR currents. It is tempting to speculate that a rapidly decaying enhancement of release probability (post-tetanic potentiation, PTP) is involved but the minimized, 3-TBS pattern is less than ideal for triggering PTP. The size of the facilitation is also larger than would be expected for PTP especially after a delay of 20 s.

As noted, the expression of SC LTP involves movement of AMPARs into the synaptic zone (Collingridge et al., 2004; Diaz-Alonso and Nicoll, 2021) and a consequent increase in evoked AMPAR currents (Kauer et al., 1988; Muller et al., 1988). The kinase PKMζ is thought to facilitate AMPAR trafficking (Sacktor, 2008) but it has not been linked to the initial expression of LTP. However, infusion of a PKMζ inhibitor (ZIP) at a concentration that has no effect on baseline transmission caused a dramatic reduction in the response growth during the first 2 min after 3-TBS (Figure 4D). ZIP did not influence the first response after the short TBS train, reinforcing the conclusion that facilitation at this point is due to a different form of plasticity.

The LTP model also posits that the increase in spine calcium elicited by NMDAR activation engages calcium-activated proteases (calpains) that cleave a wide variety of structural proteins known to be concentrated at adhesion junctions including synapses (Baudry and Bi, 2025). This disruption of the cytoskeleton is thought to relax constraints on the size of the spine and its embedded postsynaptic density, thereby opening the way for the influx of AMPARs into the latter. Assuming that the delayed growth of SC fEPSPs after TBS is due to receptor influx, then these arguments would predict that the early response growth will be blocked by calpain suppression. Indeed, a selective calpain I inhibitor (PD150606) completely eliminated the early growth phase of STP. The calpain results raise the question of whether some degree of reassembly is needed for the events leading to the gradual elevation of fEPSPs over the 2-min after 3-TBS. Past experiments showed that TBS quickly initiates actin filament assembly in spines and that this effect is required for the later LTP consolidation (Lin et al., 2005; Kramar et al., 2006). Surprisingly, a toxin (Latrunculin A) that selectively blocks actin polymerization (Rex et al., 2007) also produced a near complete elimination of response growth during STP (Figure 4D). Each of the three treatments (Lat A, ZIP, PD15) caused a pronounced reduction in the magnitude of LTP recorded 1 h after 3-TBS (Figure 4E). While the test compounds markedly reduced the immediate response to TBS, they left intact a considerable degree of potentiation at 2 min post-TBS. Moreover, the diminished potentiation observed in the drug groups decayed over the subsequent 10–15 min at about the same rate as LTP in control slices but, unlike the latter, continued to decay toward baseline.

These results are in accord with the hypothesis (see Figure 4B) that the recorded LTP *is comprised of (at least) two types of plasticity that overlap in time*, one that steadily dissipates over 60–90 min and a second that can be consolidated into an extremely stable form. Several studies have suggested that AMPAR conductance can be increased by subunit phosphorylation (Makino et al., 2011; Diering et al., 2016; Olivito et al., 2016); such an effect combined with expansion of the synaptic active zone would account for the pronounced potentiation seen in the STP period. Dephosphorylation would in this scenario lead to a progressive loss of one aspect of potentiation with long-term expression being maintained by stabilization of the structural adjustments.

### 3.2 Mechanisms supporting SC LTP differ with induction parameters

**Figure 5:**
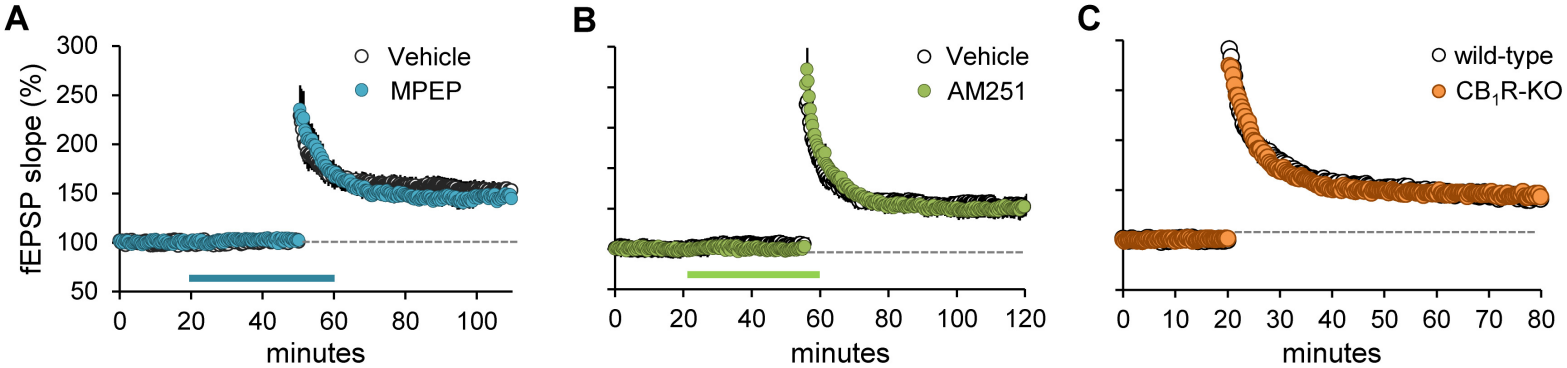
Compounds reported to disrupt CA1 LTP induced by supra-threshold stimulation (HFS or spike timing) do not attenuate LTP triggered by a single TBS train. Results are shown for antagonists of **(A)** mGluR5 (MPEP, 40 μM) and **(B)** the endocannabinoid CB1 receptor (AM251, 5 μM) and for **(C)** responses in CB_1_R knockout mice. Data modified from Wang et al. (2016b).

The above sections described substrates for, and properties of, SC LTP largely as induced by single TBS trains, including instances with as few as 2 or 3 theta bursts. As summarized in Table 1, studies using other induction protocols have obtained somewhat different results indicating that the mechanisms engaged differ with the form of inducing stimulation. LTP induced using long trains of theta bursts (Kouvaros and Papatheodoropoulos, 2016), repeated but closely spaced TBS trains (Morgan and Teyler, 2001) or second(s) long periods of HFS (i.e., ≥100 Hz) (Papatheodoropoulos and Kouvaros, 2016) are to some degree dependent upon voltage-dependent calcium channels (VDCCs); in these instances blocking NMDAR function reduces the amplitude of LTP but some potentiation remains. This is not the case for short TBS trains in which LTP is fully blocked by competitive NMDAR antagonists (Morgan and Teyler, 2001; Kouvaros and Papatheodoropoulos, 2016; Wang et al., 2016b). Similarly, potentiation induced using extended or repeated TBS trains or HFS is reliably reduced, but not eliminated, by antagonists of group 1 metabotropic glutamate receptors (mGluRs) (Kouvaros and Papatheodoropoulos, 2016; Latif-Hernandez et al., 2016). The same compounds have no detectable effects on LTP produced by a short TBS train (Wang et al., 2016b) (Figure 5A).

**Table 1:** LTP induction protocols in SC differentially recruit receptors and signaling factors.

Signaling Component	“Short” TBS	“Long” TBS	HFS	Spike-timing
NMDAR	Yes (Larson and Lynch, 1988; Kouvaros and Papatheodoropoulos, 2016)	Yes (Kouvaros and Papatheodoropoulos, 2016)	Yes (Collingridge et al., 1983; Papatheodoropoulos and Kouvaros, 2016)	Yes/no (Nishiyama et al., 2000; Cepeda-Prado et al., 2022)
VDCC	No (Morgan and Teyler, 2001; Raymond and Redman, 2006)	Partial (Kouvaros and Papatheodoropoulos, 2016)	Yes (Papatheodoropoulos and Kouvaros, 2016)	Yes/no (Tigaret et al., 2016; Cepeda-Prado et al., 2022)
mGluR5	No (Wang et al., 2016b; Koek et al., 2024a)	Partial (Shalin et al., 2006; Kouvaros and Papatheodoropoulos, 2016)	Yes (Francesconi et al., 2004; Neyman and Manahan-Vaughan, 2008; Papatheodoropoulos and Kouvaros, 2016)	Yes (Kwag and Paulsen, 2012)
mGluR1	No (Koek et al., 2024a)	Yes (Koek et al., 2024a)	Yes (Neyman and Manahan-Vaughan, 2008)	Yes (Foster et al., 2018)
CB1R	No (Wang et al., 2016b; Silva-Cruz et al., 2017)	Yes (Wang et al., 2016b; Silva-Cruz et al., 2017)	No (Terranova et al., 1995)	Yes (Noriega-Prieto et al., 2025)
BDNF	Yes (Kang et al., 1997; Chen et al., 2010b)	Yes (Kang et al., 1997)	No (Kang et al., 1997; Chen et al., 2010b)	Yes/no (Edelmann et al., 2015; Cepeda-Prado et al., 2022)
PKA	No (Rodrigues et al., 2021)	No (Park et al., 2016; Rodrigues et al., 2021)	Yes (Matthies and Reymann, 1993; Blitzer et al., 1995)	Not tested

“Short” theta-burst stimulation (TBS): single train, ≤ 10 TBS, “long” TBS: >10 TBS or multiple TBS trains; high-frequency train (HFS), spike-timing: spike-timing dependent LTP. Voltage-dependent calcium channels (VDCC), cannabinoid receptor 1 (CB1R), 2-arachidonoylglycerol (2-AG), protein kinase A (PKA). Yes = required for LTP (or major contribution); no = not required for LTP.

Also of interest is the evidence that expression of HFS-induced SC LTP can entail heightened release probability (Schulz, 1997) and, relatedly, is influenced by endocannabinoids. The latter lipid messengers are synthesized within dendritic spines and then diffuse to cannabinoid type 1 receptors (CB_1_Rs) on both juxtaposed axon terminals and astrocytes in the surrounding area (Castillo et al., 2012; Jung et al., 2012; Piomelli, 2014) with the latter eliciting gliotransmitter release (Navarrete and Araque, 2010; Noriega-Prieto et al., 2025). This endocannabinoid-driven glial transmission can influence SC terminals and, thus, the amplitude of SC LTP even though these lipid messengers are not required for its induction. In contrast, the robust and stable SC LTP produced by a single, short TBS train is not detectably affected by antagonism or knockout of the endocannabinoid CB_1_R (Figures 5B, C). A final striking example of difference between TBS- and HFS-induced SC LTP comes from early work showing that the former requires released BDNF whereas the latter does not (Chen et al., 2010b).

Spike timing-dependent LTP (t-LTP) protocols were devised to mimic activities in the hypothetical Hebb synapse wherein synaptic strengthening arises from co-activation of presynaptic and postsynaptic elements. Accordingly, in these paradigms the postsynaptic cell is depolarized to trigger action potentials and the temporal relationships between spiking and afferent stimulation are adjusted to test the prediction that a temporally tight input/spike relationship will enhance the strength of the underlying synaptic contact. Experimental work has confirmed the Hebbian predictions that induced-spiking prior to afferent stimulation reduces synaptic strength whereas spiking 10–20 ms after input activation potentiates the contact (Feldman, 2012; Kwag and Paulsen, 2012; Brzosko et al., 2019) over the course of 15 to 20 pairings (Navarrete and Araque, 2010). This experimental approach is attractive for computational neuroscientists and for investigators seeking to provide quantitative models that integrate mechanisms underlying both synaptic depression and potentiation (see (Brzosko et al., 2019) and (Feldman, 2012) for review). Evidence that the strength of t-LTP is positively correlated with the number of pre-post synaptic pairings (Noriega-Prieto et al., 2025) is seen by some as supporting the real-world relevance of this form of LTP.

However, it is becoming increasingly clear that mechanisms underlying t-LTP differ from those described above for TBS-induced potentiation. The former depends on VDCCs as well as group I mGluRs (Kwag and Paulsen, 2012) (Table 1), albeit with variations across brain regions (Araque and Noriega-Prieto, 2025). Moreover, recent work has called into question the extent to which the t-LTP is synapse-specific (a defining feature of TBS-driven LTP) and suggests that astroglia and gliotransmission may be critically involved. Specifically, a series of detailed studies by Araque and colleagues found that postsynaptic depolarization leads to endocannabinoid-, and specifically anandamide-dependent activation of CB_1_Rs and increases in calcium levels in astrocytes (Navarrete and Araque, 2010; Gomez-Gonzalo et al., 2015; Noriega-Prieto et al., 2025). This calcium increase stimulates the release of glial transmitters that mediate increased neurotransmitter release probability by nearby neurons (as noted above). The latter effect was not found to be expressed by synapses with the depolarized cell (which instead exhibit an endocannabinoid-dependent, depolarization-induced suppression of excitation, or DSE) but was instead localized to “heteroneuronal” synapses (Gomez-Gonzalo et al., 2015) that are located some distance from the manipulated cell (Navarrete and Araque, 2010; Noriega-Prieto et al., 2025). Moreover, this arrangement suggests that to electrical coupling of concatenated glial cells would further extend the spatial domain of heteroneuronal, endocannabinoid-dependent t-LTP. If these broad statements are further corroborated, the results would indicate that the nature of t-LTP associated synaptic modifications, and the mechanisms that produce them, are strikingly different from those underlying SC-LTP induced by a single short TBS train.

## 4 Contributions of sex to the diversity of mechanisms supporting SC LTP

### 4.1 Females but not males use synaptic estrogen to induce SC LTP

It has been known for some time that treatment with exogenous estrogen reversibly increases SC fEPSPs in both sexes (Foy et al., 1999; Kramar et al., 2009; Wang et al., 2016a) but early tests of the mechanisms involved have produced inconclusive results, in part due to uncertainty as to whether membrane or nuclear estrogen receptors were involved. The situation was clarified with the discovery that treatment of adult rat hippocampal slices with estradiol (E2) or selective agonists for estrogen receptor ß (ERß), but not for ERα, activated key intermediaries in postsynaptic F-actin signaling cascades involved in the consolidation of TBS-induced LTP (Kramar et al., 2009). Specifically, E2 infusion activates RhoA GTPase signaling (RhoA > ROCK > LIMK > cofilin) and actin polymerization at SC synapses in both sexes (Kramar et al., 2009; Wang et al., 2018b). Woolley and colleagues determined that protein kinase A, which phosphorylates LIMK (Nadella et al., 2009), is activated by E2 infusion in females only (Jain et al., 2019). Blocking either ROCK activity or actin polymerization *fully disrupts E2-induced SC response enhancement* indicating that effects on actin remodeling are critical (Kramar et al., 2009; Wang et al., 2018b).

These findings led to the idea that bath applied E2 promotes transient increases in spine F-actin and a weak and reversible form of LTP (Jain et al., 2019). Since hippocampal neurons synthesize estrogen (Fester et al., 2011; Hojo and Kawato, 2018) and estrogen receptors are concentrated at CA1 synapses in both males and females (Shughrue et al., 1997; Mitterling et al., 2010; Waters et al., 2015; Wang et al., 2018b), endogenous estrogen was expected to make similar and significant contributions to activity-induced LTP in both sexes. However, in hippocampal slices inhibition of P450 aromatase, the rate-limiting enzyme in E2 synthesis, fully abolishes TBS-induced CA1-LTP *in female mice only* (Vierk et al., 2012). Moreover, these sex-specific effects were replicated in cultured hippocampal slices, thus in a context lacking circulating steroids, and showed that LTP was restored by estrogen treatment. These seminal studies provided the first strong evidence that there is sex-linked diversity in the induction mechanisms for SC LTP and, in particular, that females but not males use locally synthesized estrogen to generate potentiation.

**Figure 6:**
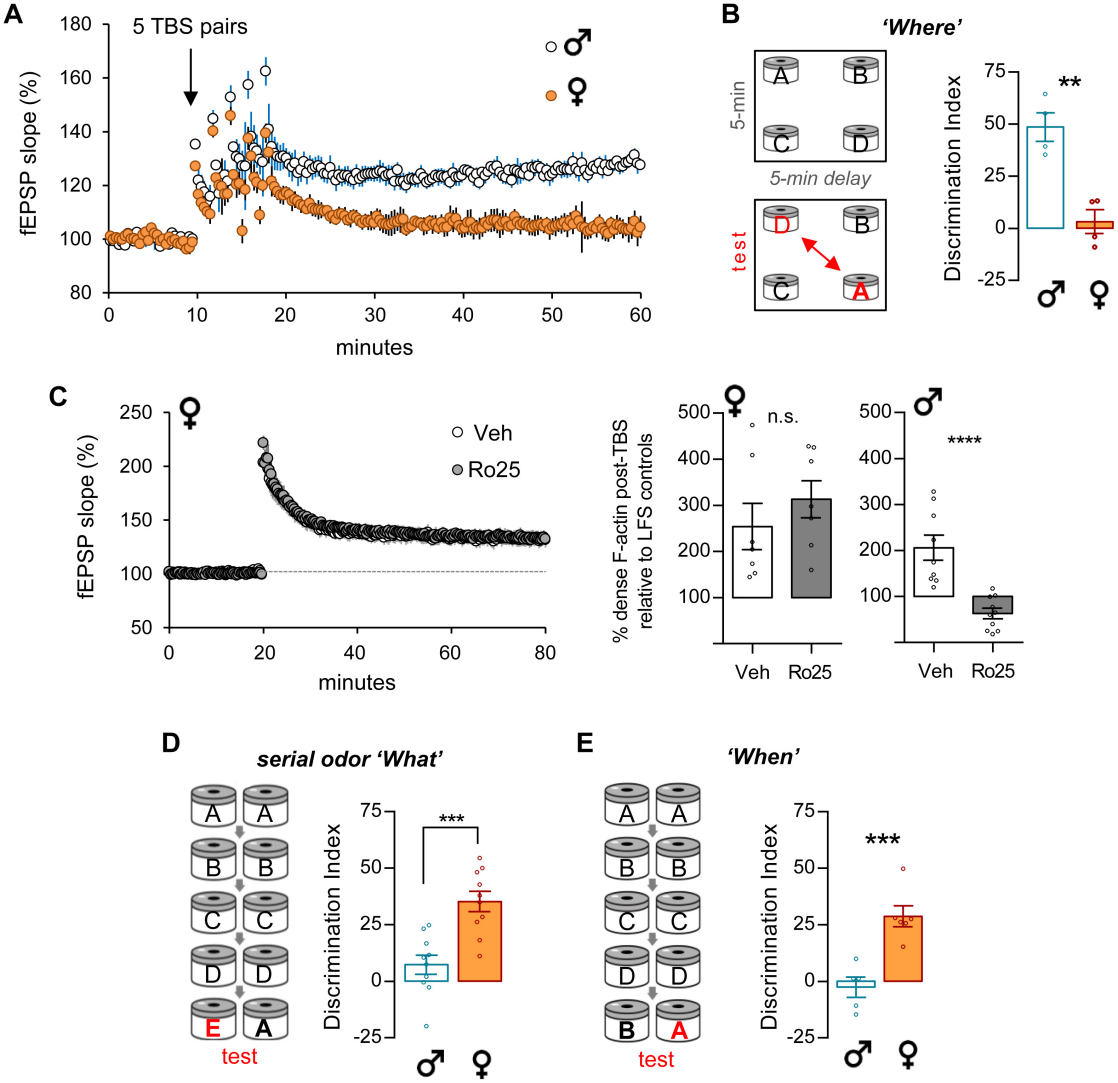
Sex differences in the threshold for SC-LTP and episodic memory. **(A)** LTP was induced using threshold stimulation (5 TBS pairs, 2 min apart) of SC-CA1 projections in slices from adult rats: in males this stimulation elicits robust and stable LTP, whereas in females initial increases in fEPSP amplitude gradually decline to baseline levels. **(B)** Mice were tested for episodic “Where” encoding in a task involving exposure to 4 distinct odors for 5-min followed by a test exposure with locations of two original odors swapped: males discriminated the novel-location odors whereas females did not. **(C)** Using near threshold TBS that effectively induced LTP in both sexes, treatment with the GluN2B antagonist, Ro25-6981 (Ro25) did not affect the magnitude of TBS-induced SC LTP (**left**) or the TBS-induced increase in spine F-actin (**middle**) in slices from females, but it did block both LTP and increases in F-actin (**left**) in slices from males. **(D, E)** Females achieve better scores in episodic “What” and “When” memory tasks. Episodic “What” task: after exposure to a sequence of 4 distinct odor pairs, mice were presented with one of the initial odors and a novel odor; at testing females discriminated (preferentially explored) the novel odor whereas males did not. **(E)** Episodic “When” task: after exposure to a cue sequence, at testing females discriminated the least recently presented odor whereas males did not. **(A)** Modified from Wang et al. (2018b); **(B–E)** Modified from Le et al. (2024a).

The sex differences in estrogen dependency appear to have important consequences for LTP expression. Although the magnitude of SC LTP is reportedly comparable in males and females receiving a 10-TBS train (Qi et al., 2016; Wang et al., 2018b), “near threshold” TBS regimens (Le et al., 2022a) elicit robust SC LTP in males but not females (Figure 6A) (Wang et al., 2018b). Given the critical role played by hippocampal field CA1 in spatial learning, these results may explain the much-cited male advantage in the encoding of cue locations (Herlitz et al., 1997; Asperholm et al., 2019). In line with this, males exhibit higher scores in tests for acquisition of the “where” component of episodic-like memory as compared to females (tested outside proestrus in rodent work) (Figure 6B) (Tulving, 1984; Le et al., 2022a, 2024a).

Investigations into mechanisms through which locally released E2 promotes SC-LTP showed that TBS-induced actin signaling is dependent on ERα in adult female, but not male, rats and mice (Wang et al., 2018b) (Figure 3D). In hippocampal slices, infusion of an ERα antagonist blocked potentiation in females only; ERß or GPER1 antagonists had no effect in either sex (Wang et al., 2018b; Le et al., 2022a). In line with this, SC LTP was found to be severely impaired in female (but not male) transgenic mice with discrete mutations that block trafficking of ERα to the membrane (Wang et al., 2018b). The ERα antagonist did not change the magnitude or shape of theta burst responses in CA1 suggesting no major effect on shunting inhibition and NMDAR currents. However, in females only, ERα antagonism did disrupt TBS-induced activation of postsynaptic TrkB and focal adhesion kinase (an integrin intermediary) along with key elements in the actin regulatory signaling cascades (Wang et al., 2016a) (see elements with asterisks, Figure 3D). It is noteworthy that TrkB signaling is not required for E2 effects on baseline synaptic strength or RhoA activation and that, beyond the sex-specific involvement of estrogen receptors in initiating actin regulatory signaling (described here and in Section 4.2), the downstream mechanisms of LTP consolidation seem to be the same in males and females; as examples, both rely on integrin activation, RhoA signaling through ROCK, new F-actin and BDNF.

Together the above results indicate that locally produced estrogen and synaptic estrogen receptor signaling are required for TBS-induced SC LTP in females only. The requisite estrogen effects are, in rats and mice, largely mediated by membrane ERα and signaling from this receptor through the RhoA GTPase cascade that controls postsynaptic actin polymerization. Thus, in females only estrogen signaling is needed for actin-dependent aspects of SC LTP stabilization.

### 4.2 Sex differences in metabotropic NMDAR contributions to SC LTP

The above results indicate that females add an estrogen step to the series of events used by males to set in motion the machinery that stabilizes SC potentiation. However, recent findings have led to a more interesting story. As noted above, males rely on metabotropic NMDAR functions to drive the cytoskeletal reorganization needed for LTP consolidation (Le et al., 2024a). In accord with this, a selective blocker of the NMDAR GluN2B subunit (Ro25-6981) greatly reduced TBS-driven increases in spine F-actin and stable LTP in males without effects on theta burst responses. Remarkably, the same GluN2B antagonist had no effect on the TBS-induced F-actin increase in females (Figure 6C) (Le et al., 2024a). Rather, this F-actin effect relies on ERα in females. Combined, the ERα and GluN2B results show that, in females, the estrogen mechanism is not an addition to, but rather a replacement for, ion-flux independent contributions of the NMDAR to LTP consolidation. In line with this, a recent analysis of potentiation of hippocampal afferents to nucleus accumbens found that in males LTP requires NMDARs, whereas in females LTP is NMDAR-independent but, instead, relies upon VDCCs and ERα (Copenhaver and LeGates, 2024). The sex-specific involvement of NMDARs and ERα in mechanisms underlying enduring synaptic plasticity is likely to have significant consequences for the suitability of different therapeutics including the proposed use of NMDAR modulators (Hanson et al., 2020) to treat cognitive dysfunction.

These findings raise the question of whether there are relative advantages to the different metabotropic receptor operations used by males and females to drive structural changes that support LTP. Other than tests for differences in LTP threshold noted above, there have been to our knowledge no between-sex comparisons of the diverse features of SC LTP (e.g., spaced trials effects, multiple consolidation periods, relative vulnerability to erasure, etc.). However, sex differences are evident in hippocampus-dependent forms of memory and it is reasonable to expect that these reflect differences in synaptic plasticity mechanisms. An example of this is found in results obtained with the relatively simple, CA1-dependent object location memory paradigm in which non-proestrus females need more sampling time than males for encoding cue locations (Wang et al., 2018b). Striking sex differences have also been described in the somewhat more complicated encoding of episodic memories by rodents. These behavioral assays measure acquisition of the identities, locations, and temporal order (“what”, “where”, and “when” information) for a collection of cues encountered for the first time and without reward contingencies. As noted above, males perform at a higher level on acquiring “where” information. In contrast, females have higher retention scores on “what” and “when” tasks (Figures 6D, E) (Le et al., 2022a). It is important to note that males perform well on the latter tests when fewer cues are included in the sample group, suggesting that the female advantage relates to the number of items that can be held in a memory list. Finally, there are major sex differences in the degree to which the learning context influences episodic encoding: female mice exhibit good retention of “what” and “when” information if tested in either the learning environment or in a familiar environment not associated with prior cue exposure. In contrast males discriminated previously experienced cues only if tested in the learning environment (Le et al., 2024b). It is possible that sex differences in the influence of context reflect previously described differences in the extent to which female rodents attend to distant context cues [see Koss and Frick (2017) for review].

## 5 Perforant path LTP

Although the modern era of work on synaptic plasticity began with the discovery of enduring potentiation in perforant path projections from entorhinal cortex to the DG (Bliss and Lomo, 1973), the mechanisms underlying perforant path LTP have not been as extensively studied as processes underlying SC LTP. Nevertheless, it has become very clear that mechanisms of potentiation in this, the major cortical input to hippocampus, are highly unusual if not unique.

### 5.1 Lateral perforant path (LPP) LTP entails an endocannabinoid-dependent increase in neurotransmitter release and reflects microglial function

The LPP arises from lateral entorhinal cortex and forms glutamatergic spine synapses within both the DG outer molecular layer and distal CA3 str. lacunosum-moleculare to innervate the DG granule cells and CA3 pyramidal cells, respectively. In contrast to the adjacent medial perforant path (MPP) and SC innervation of CA1, the LPP contains enkephalin (Gall et al., 1981) and opioids facilitate LPP-LTP presumably by inhibiting interneurons within the DG (Bramham et al., 1988). Moreover, recent studies have shown that, in contrast to mechanisms of SC LTP, LTP in the LPP-DG projection is expressed presynaptically via an increase in evoked neurotransmitter release. As such, HFS-induced LPP-LTP does not influence the evoked NMDAR/AMPAR current ratio, a signature feature of SC LTP, but entails a decrease in paired-pulse facilitation indicating that release probability is increased in the potentiated LPP synapses (Wang et al., 2016b).

As in CA1, the induction of LPP-LTP involves NMDAR activation and increased calcium signaling in the postsynaptic (spine) compartment but, in striking contrast to processes in CA1, the actin remodeling, which is indeed critical for LPP-LTP, is localized to the presynaptic element. As such, disruption of newly formed F-actin within target DG granule cells (via intracellular infusion of latrunculin A) does not impair LPP-LTP whereas bath infusion of the toxin eliminates potentiation. The retrograde spine-to-axon terminal signaling necessitated by this arrangement proved to be the endocannabinoid 2-arachidonoylglycerol (2-AG) (Wang et al., 2016b): LPP-LTP is blocked by antagonism or knockout of the CB_1_R, inhibition of group I mGluRs and intracellular buffering of calcium in the postsynaptic granule cells and is enhanced by treatments that increase 2-AG levels. Manipulation of the production or breakdown of anandamide, the second most abundant endocannabinoid in brain (Piomelli, 2003), does not influence LPP-LTP (Wang et al., 2016b).

**Figure 7:**
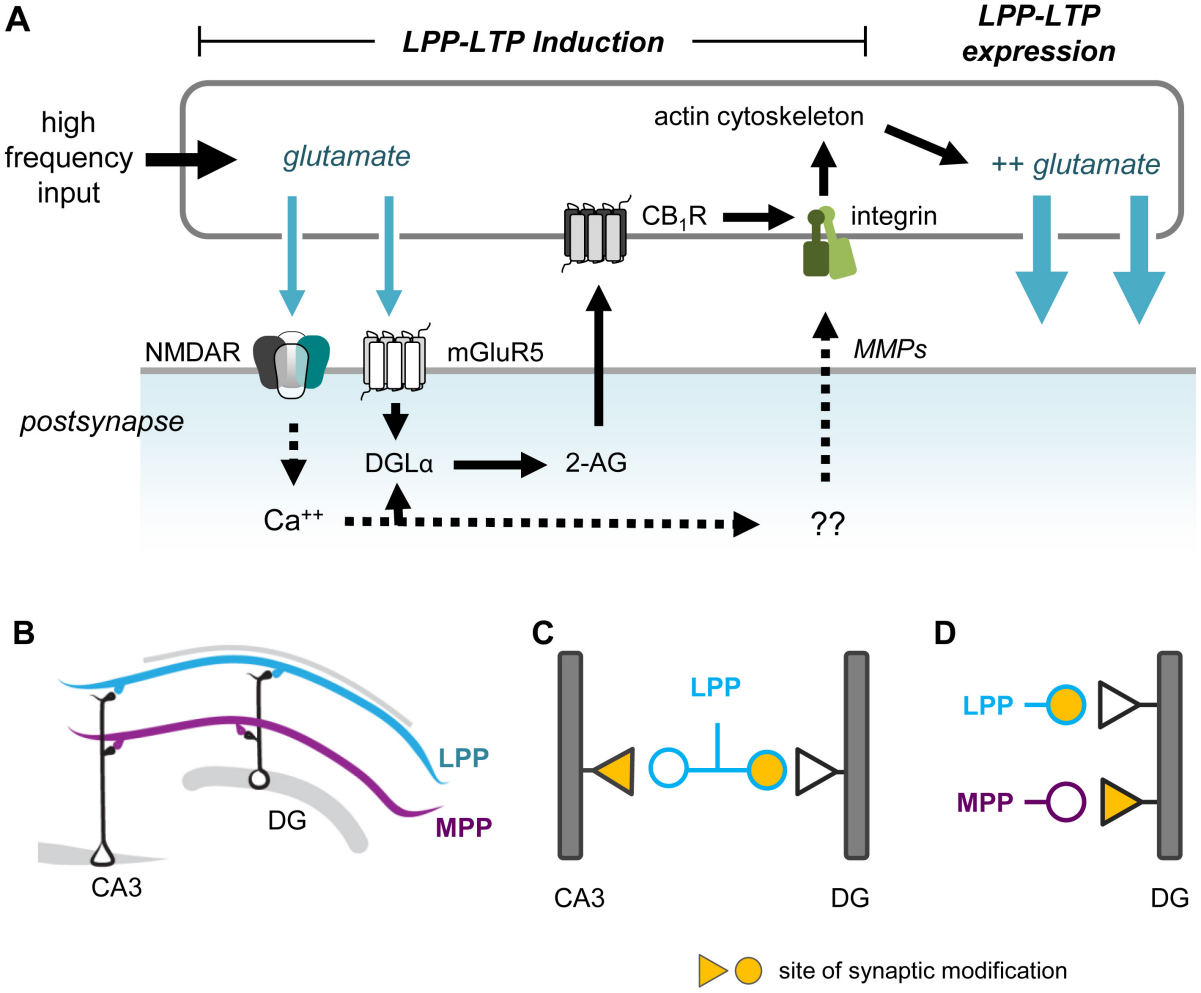
Singular substrates for LTP in the lateral perforant path (LPP) innervation of the dentate gyrus. **(A)** A substrate map for LPP-LTP. Glutamate released from the LPP activates NMDARs and mGluR5 on granule cell spines in the DG outer molecular layer. Calcium through the former and metabolic responses by the latter converge on the enzyme diacylglycerol lipase (DGLα) which synthesizes the endocannabinoid 2-AG. This product diffuses through the postsynaptic membrane to bind presynaptic endocannabinoid CB_1_Rs. At the same time extracellular matrix ligands, produced by extracellular matrix metalloproteinases (MMPs) which in this model are postulated to be activated by calcium driven release of factors from the spine (question marks) activate the presynaptic ß1 integrins. The CB_1_R and integrins coordinately signal through focal adhesion kinase to the presynaptic actin cytoskeleton and thereby effect an actin-dependent increase in evoked transmitter release that expresses LPP-LTP. **(B)** Schematic of the laminated perforant path projections to the DG granule cell and CA3 pyramidal cell dendritic fields. **(C)** LPP axons innervate the distal-most portions of both CA3 and DG dendrites. Recent work indicates that the LPP-CA3 connection uses a postsynaptic form of LTP whereas the LPP-DG connection uses presynaptic LTP expression: thus, different branches of the same axons produce pre- vs. post-synaptic forms of stable LTP. **(D)** Evidence indicates that the induction and expression of LTP at medial perforant path (MPP)-DG contacts are mediated by postsynaptic adjustments. Thus, adjacent LPP and MPP inputs on the same granule cell dendrite express different forms of LTP.

The necessary involvement of 2-AG in LPP-LTP was unexpected given that well-established functions of presynaptic CB_1_Rs are to suppress release via effects on calcium channels and vesicle-related proteins (Katona et al., 2006; Castillo et al., 2012; Piomelli, 2014) and to mediate a form of long-term depression (LTD) (Heifets and Castillo, 2009). Nevertheless, with HFS of the LPP-DG projection, 2-AG arising from the postsynaptic element engages presynaptic CB_1_Rs which act in concert with presynaptic ß1-integrins to increase presynaptic actin polymerization and elicit an enduring increase in neurotransmitter release (Figure 7A) (Wang et al., 2016b, 2018a). These effects are obtained with and without inclusion of the GABAA receptor antagonist picrotoxin in the hippocampal slice bath, demonstrating that the endocannabinoid effects on LTP are not secondary to modulation of inhibitory transmission. Although somewhat exotic for brain, the endocannabinoid > CB_1_R-integrin > cytoskeletal signaling cascade has been described for certain peripheral cells (Dalton et al., 2013; Malenczyk et al., 2013) and presynaptic F-actin is known to be critical for vesicle recycling and docking involved in release (Shupliakov et al., 2002; Brodin and Shupliakov, 2025).

The LPP does not terminate exclusively in the DG molecular layer but also generates a collateral branch that innervates the distal-most segment of CA3 pyramidal cell apical dendrites (Witter, 1993). Evidence strongly suggests that individual LPP axons contact both DG and CA3 targets (Tamamaki and Nojyo, 1993) (Figure 7B). Thus, it was surprising to find that, in contrast to properties of LPP-DG contacts, the LPP-CA3 synapses exhibit postsynaptic expression of LTP that is not reliant on CB_1_R signaling (Quintanilla et al., 2024). Thus, diverse forms of plasticity can be seen at two branches of the same LPP axons (Figure 7C).

Further differentiation between the DG and CA3 branches of the LPP became evident with the remarkable discovery that in adult mice depletion of the brain microglia population, accomplished by placing adult C57BL/6 mice on a diet including colony stimulating factor 1 receptor antagonist PLX5622 for 10 days (Green et al., 2020), profoundly impairs potentiation at LPP-DG synapses but not at LPP-CA3 contacts (Chavez et al., 2025). Work leading to this finding had demonstrated that in rats and mice exposure to cannabinoids during adolescence leads to disturbances in homeostatic microglial functions as well as impairments in LPP-LTP (Le et al., 2022b; Lee et al., 2022). To test if the microglial disturbances contributed to changes in plasticity the effects of microglial depletion were assessed. The over 90% depletion of microglia achieved with PLX5622 treatment had no detectable effect on baseline transmission at any of four sites in intrahippocampal circuitry or on potentiation in the SC or Medial Perforant Path (MPP) systems. These results underscore the singular nature of LPP-DG plasticity and the importance of both pre- and post-synaptic elements in dictating the form of LTP expressed.

### 5.2 The medial perforant path LTP mechanisms are distinct

Mechanisms of MPP LTP have not been extensively studied but available evidence indicates that they do not conform to properties of either the SC or LPP-DG systems. The MPP arises from medial entorhinal cortex and innervates granule cell dendrites in the DG middle molecular layer and CA3 pyramidal cell dendrites in str. lacunosum-moleculare; in both fields the MPP innervation is immediately subjacent to the LPP terminal field (Witter, 1993). Although technical factors preclude the simple tests for changes in MPP transmitter release probability used to localize the site of LTP in other systems (LPP, SC), results indicate that, unlike plasticity in the LPP, MPP-DG LTP is expressed by postsynaptic changes and does not rely on endocannabinoid transmission (Figure 7D; Wang et al., 2016b).

Supporting a postsynaptic locus of expression, MPP potentiation relies on NMDAR function and increases in intracellular calcium in the granule cells (Colino and Malenka, 1993; Wang et al., 2016b). Potentiation in the MPP entails increases in AMPAR binding (Maren et al., 1993) and heightened membrane GluR1 insertion (Williams et al., 2007) in DG molecular layer. Moreover, MPP-LTP reportedly involves postsynaptic signaling linked to actin cytoskeletal remodeling. *In vivo*, HFS delivered to the MPP increases F-actin in dendritic spines in the DG middle molecular layer. Moreover, similar to processes in the SC system, this actin remodeling is accompanied by increases in phosphorylated (p) cofilin, p-ERK1/2, and p-FAK within granule cell dendritic spines, and is blocked by both latrunculin A and RhoA inhibition (Fukazawa et al., 2003; Yang et al., 2003; Huang et al., 2007). MPP-LTP also relies on BDNF/TrkB function (Bramham, 2007). Together these results support the idea that MPP-LTP is triggered by postsynaptic NMDAR activation, expressed by increases in synaptic AMPARs and stabilized, at least in part, via activity-dependent changes in the post-synaptic actin cytoskeleton. These are all features of postsynaptic SC LTP in field CA1.

However, in other respects mechanisms of MPP-DG LTP deviate from those expressed by SC synapses. In the latter field, potentiation induced by TBS or HFS relies on activation of postsynaptic CaMKII and ERK1/2. However, in the MPP-DG system, inhibition of either kinase fails to disrupt LTP in adult hippocampal slices (Cooke et al., 2006; Wu et al., 2006; Lisman et al., 2012) whereas combinations of inhibitors (e.g., for CaMKII and ERK1/2) do attenuate potentiation. Moreover, while SC LTP is largely triggered by NMDAR activation but expressed by changes in AMPAR currents (Muller and Lynch, 1988; Granger and Nicoll, 2014), in the MPP-DG system, evidence suggests that potentiation involves movement of NMDARs containing the GluN2D subunit from perisynaptic regions into the synaptic active zone and expression, at least in part, by greater NMDAR currents (Harney et al., 2008).

Beyond differences in the relative importance of elements involved in potentiation across the different systems, MPP-LTP appears to be singular in the nature of involvement of BDNF. As noted above, TBS-induced SC LTP is fully dependent upon BDNF/TrkB for induction but not expression: e.g., the BDNF scavenger TrkB-Fc blocks LTP expression if it is infused during the period of TBS but not with infusion 10 min after TBS (Rex et al., 2007). Infusion of BDNF alone, absent LTP-inducing stimulation, potentiates MPP responses thereby eliciting what has been termed BDNF-LTP (Panja et al., 2014). Similar BDNF-only induction has been described by some to occur in the SC system (Kang and Schuman, 1995; Korte et al., 1998) but this was not observed by others (Figurov et al., 1996; Rex et al., 2007). Nevertheless, there is good evidence that in the MPP the neurotrophin is required for ongoing maintenance of the potentiated state. Infusion of the BDNF scavenger 2 and 4 h after the HFS-induction of LTP leads to fairly rapid MPP depotentiation. Associated findings indicate that continued BDNF-induced signaling to Map Kinase interacting Kinase (MNK) is needed to sustain translation of proteins that support LTP in this particular system.

## 6 Discussion

The above overview summarizes a now considerable body of evidence indicating that the hippocampus expresses several clearly different forms of activity-induced LTP. The SC projections to CA1 apical dendrites exhibit mechanistically distinct forms of potentiation that are induced postsynaptically with differences reflecting the nature of the inducing stimulus and sex. In contrast, the lateral perforant path (LPP) expresses a highly unusual form of LTP that is induced postsynaptically, dependent upon endocannabinoids, and expressed presynaptically by increased neurotransmitter release (Wang et al., 2016b, 2018a). Specializations of these types are commonly thought to reflect adaptations needed for functionally useful endpoints. The various forms of synaptic modification might, for example, facilitate the encoding of different types of memory or, perhaps, encoding limited to specific situations and task demands. However, it is also possible that diversity was not, in fact, a target for evolutionary pressures but instead reflects restrictions imposed by regional specializations for other basic brain operations. It is generally agreed that firing patterns in collections of neurons constitute the means whereby a given region processes an incoming signal and generates a response that can be read by the next station in a circuit. These responses can differ markedly between the nodes in a complex circuit as was found to be the case for the successive links of the network that extends from the olfactory bulb to olfactory cortex to dentate gyrus (Trieu et al., 2015) and from there through the hippocampal circuit to the CA1 output station (Gunn et al., 2025). Although it is difficult to specify computations executed by circuit nodes, recent studies have shown that the subfields of hippocampus are specialized for particular types of frequency filtering, signal amplification and reverberation activities (Cox et al., 2019; Quintanilla et al., 2022; Gunn et al., 2025). The features that enable these local operations will determine which receptor systems and biochemical pathways are available for changing synaptic strength.

Developmental changes can be useful in evaluating the possibility that prominent biological phenomena are shaped by other, perhaps more fundamental adaptations. Dendritic growth and synaptogenesis in the rat hippocampus proceed at a high rate early in postnatal life but slow dramatically in the third week and essentially stop in the fourth week (Stanfield and Cowan, 1979; Gall and Lynch, 1980; O'Kusky et al., 2000). Surprisingly little is known about the factors that terminate the growth phase, but there is evidence that levels of the active (dephosphorylated) form of the actin severing protein cofilin increase dramatically as growth slows (Lauterborn et al., 2017). This, and related observations, led to the hypothesis that the ontogenetically late increase in active cofilin suppresses the assembly of long actin filaments and thereby works against further stable elongation of dendritic processes. The idea is of interest in the present context because (1) stable CA1-LTP emerges along with dendritic maturation (Kramar and Lynch, 2003; Ostrovskaya et al., 2020), and (2) TBS causes a transient phosphorylation (inactivation) of cofilin at adult CA1 synapses and relatedly triggers actin filament assembly within spines (Kramar et al., 2006; Chen et al., 2007; Rex et al., 2010). TBS, in this argument, causes targeted spines to briefly revert to an earlier growth phase and thereby enables structural modifications that affect spine shape and size, and the potency of synaptic contacts. The adaptations needed for CA1-LTP might therefore be limited to connecting the bursts of synaptic receptor activity and calcium influx associated with TBS (or HFS) to a pre-existent complexity associated with determinant growth. Instances in which features of considerable survival value (synaptic plasticity, memory encoding) are secondary to other adaptations, rather than being a primary target of selection pressures, are not uncommon.

Whatever their origins, it is generally assumed that different forms of plasticity are needed for the encoding of the type and, perhaps, the longevity of memory. But many and indeed most LTP studies use induction parameters, such as HFS and neuronal depolarization with spike-timing, that are unlike conditions that might obtain during routine brain operations. It is of course possible that synaptic changes obtained in a well-controlled neurobiological experiment occur at a much lower threshold during learning but testing this is difficult. The enormous capacity of everyday memory strongly suggests that only a very small percentage of storage elements (synapses) are used to acquire a given piece of information. If so, then it will be extremely challenging to assess learning-related changes in the number or size of synapses expressing an LTP marker. A weaker form of the numbers problem is encountered in studies searching for the substrates of TBS-induced CA1-LTP. As mentioned, only a handful of synapses are needed to produce field EPSPs of the size recorded in a typical LTP experiment (Chen et al., 2007). But it is possible, in slice studies, to ensure that nearly all of these are localized to a specific sub-lamina of the dendritic tree and to use multiple electrodes to potentiate separate populations of synapses that converge on the same dendritic zone (Chen et al., 2007; Rex et al., 2007, 2009). Experiments using this approach, 3-dimensional reconstructions of tens of thousands of synapses in the activated terminal field and dual immunolabeling have reliably detected small but significant TBS-driven increases in the percentage of contacts containing activated actin signaling proteins (Figure 3) (Chen et al., 2007, 2010b; Rex et al., 2009; Seese et al., 2012). As discussed, blocking these signaling events, or the actin polymerization associated with them, thoroughly suppresses LTP consolidation. Studies using the same imaging strategy to assess the effects of learning (Fedulov et al., 2007; Chen et al., 2010a; Cox et al., 2014; Seese et al., 2014), then provided evidence that the actin management steps and synapse expansion that are critical for LTP also occur in conjunction with, and are needed for, the formation of everyday memories (Chen et al., 2010b; Rex et al., 2010; Babayan et al., 2012; Cox et al., 2014; Young et al., 2014).

The combination of results from studies using (i) naturalistic induction conditions (2–3 theta bursts), (ii) the occurrence of predicted (and unusual) neurochemical events during learning, (iii) sparse coding, and (iv) selective disruption by uncommon agents (e.g., neutralizing antisera against select integrin subtypes, venom derived integrin antagonists, BDNF TrkB-Fc scavengers) (Wang et al., 2008, 2016a, 2018b; Chen et al., 2010b; Babayan et al., 2012) makes a reasonable case for field CA1, SC LTP as a memory substrate. In line with this, similar synaptic signaling cascades and actin remodeling have been identified in association with synaptic plasticity, and related learning, in the amygdala (Lamprecht, 2014). Evidence of this kind is lacking for other forms of plasticity found in the hippocampus in large part because their substrates are not as well understood as those for TBS-induced CA1-LTP. This precludes testing if learning increases, in predicted subfields, synaptic markers associated with the plasticity variant under study.

The diversity of LTP is most evident in aspects—induction and expression—that do not readily translate to behavior. Models can be useful in this regard. For example, inserting TBS-induced LTP into simulations of a simple version of association cortex resulted in a system that was remarkably efficient in generating hierarchical categorization of environmental cues (e.g., plant > flower > rose) (Ambros-Ingerson et al., 1990). In this instance, storage according to LTP rules (as expressed in CA1) enabled vital but complex computations in addition to providing for cue recognition. Other work obtained evidence for the functional utility of LTP results obtained in experiments using sequential activation of small CA3 inputs to single CA1 pyramidal cells. These studies found that the order in which the inputs were activated with TBS dictated the degree to which each afferent increased its synaptic strength (Larson and Lynch, 1989). Implementing these LTP rules into CA1 simulations produced a network that learned extraordinarily large numbers of full-length words with minimal errors (Granger et al., 1994). The above simulation results were entirely dependent on the characteristics of TBS-induced CA1-LTP—substituting variables from other forms of plasticity such as presynaptic expression of LTP would strongly affect categorization and capacity. However, it is not unlikely that LPP-LTP and the other forms of plasticity would support different types of computation.

Models can also serve to identify higher order consequences of adding seemingly arbitrary features to basic systems. While LTP threshold is associated with sex differences in episodic learning (Koss and Frick, 2017; Wang et al., 2018b; Le et al., 2022a, 2024a), it also has a profound effect on the size and number of categories constructed by network simulations. Simulations using higher LTP thresholds require a greater similarity between cues for their inclusion into a category than is the case with inclusion of more easily induced LTP. The combination of experimental results and modeling therefore leads to the rather startling prediction that, due to differences in LTP threshold, male and female rodents build different hierarchical classification systems in their respective worlds. If these results generalize across the mammals, then they would point to substantial sex differences in the prominent roles played by hierarchies in human cognition (Frank et al., 2023).

A number of studies have shown that excessive activation of the signaling cascades used to adjust synaptic strength can also produce neuropathology (Chan and Mattson, 1999; Amini et al., 2013; Chen et al., 2016; Dore et al., 2016). If so, then the sex differences in LTP-related signaling could contribute to sex-specific responses to insult and conditions associated with neurodevelopmental disorders. As described above, TBS-induced SC LTP requires the involvement of synaptic estrogen receptors in females but not in males. This reliance on estrogen receptor signaling created a vulnerability to effects of cannabinoid exposure. Specifically, we conducted experiments investigating potential enduring effects of exposure to tetrahydrocannabinol (THC), the primary psychoactive compound in cannabis, on LTP and episodic encoding. In both rats and mice, daily treatments with THC over the ages of puberty (postnatal days 30–43) disrupted adult SC-LTP in females but had no measurable effect in males (Le et al., 2022b). This sex-specific impairment was accompanied by a loss of estrogen signaling at CA1 synapses. Thus, it seems that the addition of estrogen receptor involvement to the complex processes underlying synaptic modifications for SC-LTP introduced a female vulnerability to the use of a recreational drug during adolescence (Le et al., 2022b). The differential effects of THC on SC LTP had predicted consequences for learning: THC-treated females exhibited impaired performance in spatial memory tasks and episodic “what” encoding. The only defect evident in THC-treated males was a reduced retention score in the episodic “what” paradigm in mice but not rats (Le et al., 2022b).

The effects of early life THC exposure also reflect mechanisms underlying regional differences in LTP. In mice, exposure to THC during adolescence (in the above paradigm) disturbs both homeostatic activities of microglia (Lee et al., 2022) and, as discussed above, potentiation of the LPP-DG connection which has a singular dependency upon these glial cells (Chavez et al., 2025). These points suggested that adolescent THC exposure may impair LPP-LTP and this prediction was confirmed with evidence for a severe reduction in the magnitude of LPP-LTP in males and females tested in adulthood. The adolescent-THC treated mice also had impaired performance on a battery of LPP-dependent episodic memory tasks (Le et al., 2022b). These findings further suggest that normalization of microglial function might restore LPP-LTP and forms of learning that rely on this system.

Taken together the studies of cannabinoid effects provide examples of how an influence or insult, in this case drug usage, can interact with sex-specific and regional differences in mechanisms of LTP to produce substantial changes in the functional properties of circuitry required for the acquisition of episodic memories and, thus, higher cognitive function.
